# Phytochemical Analysis and Pharmaceutical Applications of Monoterpenoids Present in the Essential Oil of *Boswellia sacra* (Omani Luban)

**DOI:** 10.1155/adpp/3536898

**Published:** 2025-02-24

**Authors:** Foziya Khan, Luay Rashan

**Affiliations:** Research Center, Biodiversity Unit, Dhofar University, Salalah, Oman

**Keywords:** *Boswellia*, diterpenes, essential oil, frankincense, monoterpenes, phytochemicals

## Abstract

Due to its intricacy and long-term usefulness, traditional medicine continues to be practiced in several nations. Among the many medicinal plants found in the Dhofar region of Oman, the aromatic oleo-gum resin generated by *Boswellia sacra*, commonly referred to as frankincense, stands out for its medical and commercial significance. Resin-carrying ducts are unique to members of the *Boswellia* family. *Boswellia sacra* Flueck is one of the 29 species in the genus *Boswellia* (Burseraceae) and has long been cultivated for its aromatic gums and resins for use as incense. In addition to the resins (60%–80% alcohol soluble), gums (15%–20% water soluble), and essential oil (5%–7%), other components, including polysaccharides and polymeric compounds, also exist in smaller amounts. Physiochemical analyses indicate that *Boswellia* resin oil is made up of 42.5% diterpenes, 13.1% monoterpenes, and 1% sesquiterpenes. Traditional medicine makes extensive use of frankincense for the treatment of stomach diseases, Alzheimer's disease, and hepatic illnesses. The bioactive chemicals present in frankincense, particularly boswellic acids, are plentiful. The current review examines various compounds present in different species of *Boswellia*, especially *Boswellia sacra*, along with their structure.

## 1. Introduction

Frankincense and olibanum both come from trees and bushes. Several tree species in the genus *Boswellia* and family Burseraceae secrete this oleo-gum resin through special ducts in their bark. The trees are small with compound leaves and papery bark. *Boswellia sacra* is a shrub or tree up to 5 m high, can be single- or multistemmed, and has dense interwoven branches with clusters of leaves at the ends. *Boswellia sacra* also belongs to the Burseraceae family, which is composed of approximately 700 species and 20 genera. Its blossoms are star-shaped [1, 2]. Frankincense derives from the Old French phrase franc encens, meaning “pure incense.” It is known as luban in Arabic, which means “white” or “cream,” libanos in Greek, and etan in Ethiopian [3–5]. Olibanum (frankincense) has traditionally been burned as incense [6], but more recently it has been used in the production of cosmetics and perfumes. Other medicinal uses for olibanum can be found in both Unani (Islamic) and Chinese traditional medicine, primarily due to its anti-inflammatory, sedative, antihyperlipidemic, and antibacterial properties [7–9].

The Dhofar region in the Sultanate of Oman is home to *Boswellia sacra* trees [10]. The resin produced by the ducts on the leaves of certain plants belongs to the genus *Boswellia*. Although this genus comprises 29 species, only *Boswellia sacra* Flueck is known to provide the aromatic gums and resins used in incense. This native species of the Arabian Peninsula can only be found in the southwestern part of Oman and in the Hadramawt and Mahra districts of Yemen [11]. In addition to Arabia, the Horn of Somalia is the most important location for producing gum frankincense known as “bejo” [12].

### 1.1. Chemical Composition of *Boswellia* Species

Approximately 60%–80% of the resin from *Boswellia* species is alcohol-soluble, 15%–20% is water-soluble gum, 5%–7% is essential oil (EO), and there are lesser amounts of polysaccharides and polymeric compounds. Numerous kinds of active phytocompounds are present in *Boswellia* plants. Species diverge due to variations in the amount and quality of these compounds. Climate, harvest season, and geographical conditions all contribute to these variations [13].

In 1840, Stenhouse studied the components of frankincense essential oil (FEO) and found that, depending on the origin of the resin, there can be as many as fourteen different monoterpenoid compounds, including pinene, dipentene, phellandrene, and cadinene [14]. Tschirch and Halbey initially described boswellic acid, the acidic component of olibanum (C_32_H_52_O_4_), in 1898. However, it was too early to determine the precise framework [15]. In 1932, Winterstein and Stein [16] conducted a comprehensive study of olibanum resin. Later, in the 1960s, various derivatization techniques revealed other boswellic acids, including *α*- and *β*-boswellic acids, 3-O-acetyl-11-hydroxy-*β*-boswellic acid, and 11*α*-hydroxy-*β*-boswellic acid. In 1967, Snatzke and Vértesy [17] described the structure of acetyl-11-keto-*β*-boswellic acid (AKBA) for the first time. In 1978, Pardhy and Bhattacharya [18] isolated the following compounds from *B. serrata* Roxb.: *β*-boswellic acid (BA), acetyl-*β*-boswellic acid (ABA), 11-keto-*β*-boswellic acid (KBA), AKBA, tirucallic acids, and a diterpenoid cembrene–based alcohol termed “serratol.” In 1977, Obermann [19] utilized the gas chromatography–mass spectrometry (GC–MS) method to conduct the first in-depth analysis of olibanum EO from several regions. In 1984, Maupetit published a comprehensive assessment of the “Aden” variety of olibanum, recording 47 newly discovered compounds in both the resin and the oil, in addition to the 169 previously described chemicals and the pyrolysis products [20]. The most recent research on *B. serrata* oil, along with a study by Humprey et al. that compared *B. carterii* oil with that of rosemary, cumin, and ginger, largely comprised re-examinations of established facts. These investigations highlighted the challenges in tracing the origins of olibanum resin and establishing a consistent olibanum oil standard [21]. More recent research uncovered sesquiterpenes, alcohols, esters, and boswellic acids in an ether-soluble gum fraction containing 3%–8% oil. However, polysaccharides accounted for 25%–30% of the ether-insoluble gum component [22]. Diterpenoids are another class of nonvolatile compounds [23].

Physiochemical studies have revealed that *Boswellia* resin oil is composed of 42.5% diterpenes, 13.1% monoterpenes, and 1% sesquiterpenes. Other major components are octyl acetate (13.4%), duva-3,9,13-trien-1,5alpha-diol-1-acetate (21.4%), o-methyl anisole (7.6%), thunbergol (4.1%), sclarene (2.9%), alpha-pinene (3.1%), 9-cis-retinal (2.8%), verticiol (1.2%), octyl formate (1.4%), decyl acetate (1.2%), naphthalene decahydro-1,1,4a-trimethyl-6-methylene-5-(3-methyl-2-pentenyl) (5.7%), and n-octanol (1.1%) [24–30].

The volatile oil contains various terpenes, including monoterpenes (in hydrocarbons, alcohols, and ketonic forms) and sesquiterpenes, along with small amounts of diterpenes. The composition of the oil varies according to its environment, harvest, and geographical origin [31]. Numerous boswellic acids have been extracted from the frankincense resin of Indian, Arabian, and Somalian species of *Boswellia* [32–34]. Predominant compounds present in the EO of different *Boswellia* species are listed in Table 1.

### 1.2. EO of *Boswellia serrata*

Approximately 45% of the EO of *Boswellia serrata* is composed of a compound called *α*-pinene, the hydrodistillate of which produces clear oil. This oil is primarily composed of methyl chavicol (11.6%), *α*-thujene (12%), *α*-pinene (8%), sabinene (2.2%), myrcene (3.8%), methyl eugenol (2.1%), *α*-phellandrene (1%), limonene (1.9%), cembrenol (1.9%), *β*-pinene (0.7%), linalool (0.9%), p-cymene (1%), germacrene D (2.0%), cembrene A (0.5%), perillene (0.5%), and kessane (0.9%). The monoterpene 5,5-dimethyl-1-vinylbicyclo-hexane (2%), along with the diterpenoid components p-camphorene (0.3%) and m-camphorene (0.7%), has also been found [27, 35, 36]. In the resinous portion of *B. serrata,* several monoterpenes, diterpenes, and triterpenes, along with tetracyclic triterpenic acids and four important pentacyclic triterpenic acids (AKBA, KBA, ABA, and BA), are present, which inhibit the proinflammatory enzymes. AKBA is especially effective in blocking the inflammatory enzyme. *α*-Pinene, 5-lipoxygenase, limonene, and *β*-caryophyllene are the primary components of both *B. carterii* and *Boswellia sacra*. Among the volatile components present in *B. serrata* extract, *α*-thujene (11.7%) is the most abundant [37, 38].

### 1.3. EO of *Boswellia carterii*

*B. carterii* EO is one of the most widely researched olibanum oils. Various classes of compounds present in the EO, as reported by Borotova et al. [39], are listed in Table 2. Octyl acetate is the most abundant, making up 60% of the resinous oil. Light yellow in color, the prime components of *B. carterii* hydrodistillate oil have been identified as *α*-pinene (10.9%), *α*-thujene (1.7%), p-cymene (1.4%), E-*β*-ocimene (1.7%), camphene (1.0%), *β*-pinene (0.7%), sabinene (0.7%), myrcene (0.5%), Z-*β*-ocimene (0.4%), hexyl acetate (0.3%), limonene (1.5%), 1,8-cineole (1.2%), octyl acetate (39.3%), linalool (2.1%), trans-Verbenol (0.4%), bornyl acetate (2.2%), *α*-pinene epoxide (0.5%), terpinen-4-ol (0.4%), cembrene A (2.1%), E-nerolidol (0.2%), geranyl acetate (0.4%), cembrene C (0.1%), 7,11-triene (6.0%), incensole (1.0%) verticilla-4 (20), incensole acetate (2.3%), and 1-octanol (11.9%). Diterpenoid compounds and octyl acetate have also been discovered in the n-hexane extract of *B. carterii*, with octyl acetate and monoterpenoid constituents detected in lower concentrations [24, 35, 40].

### 1.4. EO of *Boswellia sacra*

An analysis of 78 Mughsayl frankincense and 10 Hasik frankincense EO samples revealed that unlike the higher priced Hasik frankincense, which is rich in aromatic myrcene and limonene, Mughsayl frankincense has a larger concentration of *α*-pinene. This discrepancy accounts for the widely praised citrusy aroma of Hasik resin. The scents of *Boswellia sacra* resins vary slightly depending on where they grow [41]. Chemotypes is a term used to describe chemical profiles of the same species [42, 43] that vary according to the soils, climate, moisture, and geography. In a recent study, researchers compared the EO of *B. serrata* (India), *B. carterii* (Somalia), and *Boswellia sacra* from four different regions in Oman. They found that Omani EOs were unique due to their essential component being *α*-pinene, which can therefore be used as a chemotaxonomical marker to determine the exact location of origin [44]. The mean optical rotation (+30°) for Mughsayl EO, which contains 81% *α*-pinene on average, is greater than that (+19°) for Hasik EO, which contains 60% *α*-pinene on average. The unique scents of *Boswellia sacra* and *B. carterii* resins make them easily distinguishable. The odor of (+)-α-pinene is reminiscent of minty turpentine, whereas that of (−)-*α*-pinene is akin to that of turpentine pine [41, 45]. Fresh citrus (lemon/orange) is conveyed by the other primary component, (+)-limonene, whereas its chiral companion, (−)-limonene, has a more turpentine-like perfume with a lemon undertone [45, 46]. Thus, the turpentine mint and lemon scents in *Boswellia sacra* resin are more pronounced than in *B. carterii* resin, whereas the turpentine pine and lemon aromas are more predominant in *B. carterii* resin. In monoterpene biosynthesis, the variation in enantiomeric pair ratios of monoterpenes between resins from the Arabian *Boswellia sacra* and the African *B. carterii* is attributable to the differential expression of chiral-specific enzymes. The geographical isolation caused by the Red Sea Rift Valley between the Arabian Peninsula and East Africa is the main reason for the genetic shift and speciation of the *B. carterii* tree in East Africa and *Boswellia sacra* tree in Arabia [41].  Table 3 compares the compounds present in the *Boswellia sacra* collected from Mughsayl and Hasik areas, as reported by Woolley et al. [41].

## 2. Monoterpenes in *Boswellia sacra*

**Figure 1:**
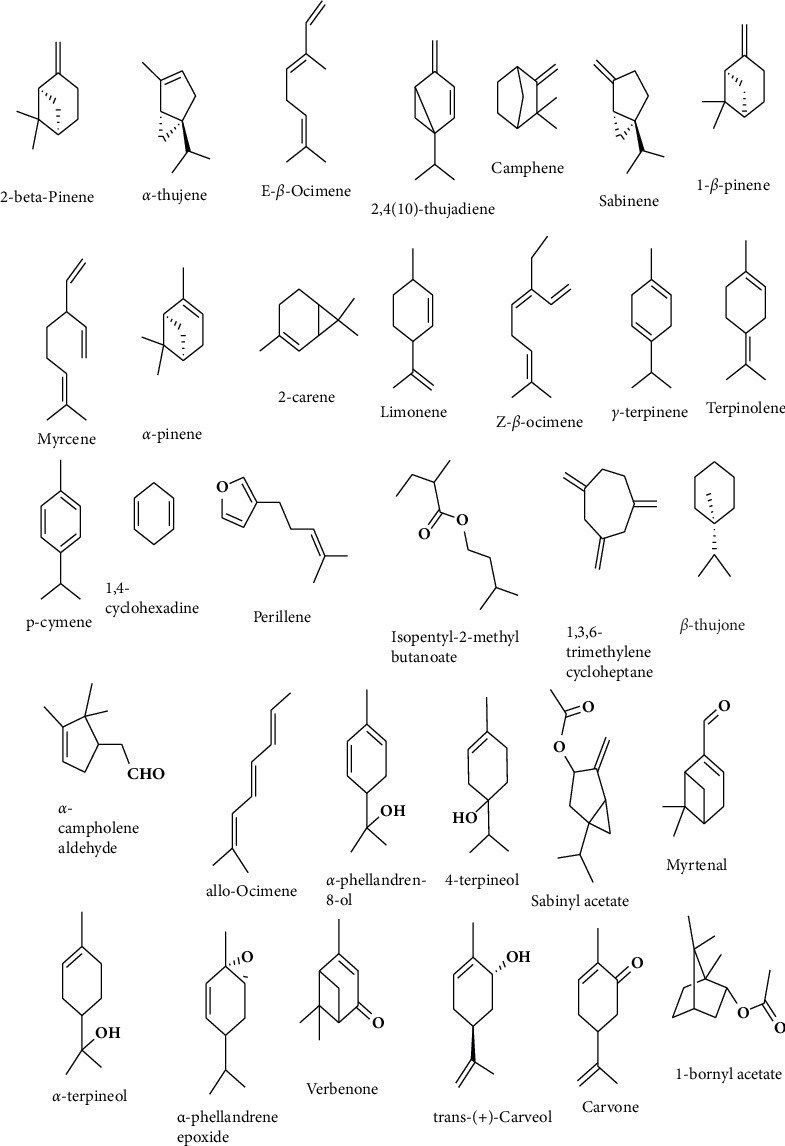
Structure of monoterpenes from the FEO of *B. sacra* resin [10].

Cembrenol, viridiflorol, dimethyl ether orcinol, and, most notably, incensole are constant, reliable identifiers of *B. carterii*. Higher levels of *α*-pinene and delta-3-carene are characteristic of *Boswellia sacra*, whereas higher levels of myrcene, limonene, *α*-thujene, germacrene D, *trans*-*β*-caryophyllene, incensole, and unknown (RI 2175) are typical of *B. carterii*. Preliminary tests carried out by Woolley et al. [41] revealed no major changes in quality or quantity between the two species. The literature reports widely varying concentrations of *α*-pinene (5%–75%) in FEO available for sale worldwide [32, 36, 44]. Sacks of frankincense resin preserved for almost 2 years were located by Wooley et al. [41] in commercial regions in Ethiopia, Yemen, Somalia, and Oman. The majority of GC–MS compositions reported in the literature may originate from storage or aged (2–5 years) frankincense resin, as their data generally indicate at least 50% *α*-pinene for *B. carterii* and up to 80% *α*-pinene for *Boswellia sacra* where resins were distilled within a year of collection. These findings are consistent with those of recently published studies on Omani *Boswellia sacra* resin collected in the Dhofar region [44]. Appropriate characterization of the composition of frankincense resins is facilitated by the knowledge that circumstances and duration of storage for commercial frankincense resin contribute to the fluctuation of monoterpene content. However, age-related *α*-pinene variability makes it difficult to distinguish between *B. carterii* and *Boswellia sacra* resins using conventional GC–MS alone [41]. Structures of various monoterpenes found in *Boswellia sacra* are presented in Figure 1 [10].

A case study demonstrated that *Boswellia sacra* resin, when taken orally, protects against urothelial carcinoma [47]. Volatile monoterpenes such as myrcene, limonene, *p*-cymene, *α*-pinene, and boswellic acid are responsible for *Boswellia sacra* oil (BO) antitumor activity [48]. FEOs derived from *Boswellia sacra* exhibit promising responses to many cancer cells, but their therapeutic utility is limited by hydrophobicity, poor absorption, and nonselective transport to tumor cells [49]. Analysis of BO through GC–MS revealed the presence of 32 distinct chemicals. EO compositions were recognized, identified, and characterized through mass spectral comparisons with the National Institute of Standards and Technology (NIST) database. According to the GC–MS results, *α*-pinene accounts for 61.05% of the BO, whereas D-limonene accounts for only 9%. GC–MS analysis also revealed the presence of monoterpenes (88.51%), esters (2.1%), oxygenated monoterpenes (8.78%), and sesquiterpenes (0.35%) in BO, as shown in Table 4 [50].

The chemical components of FEO allow for easy discrimination between the numerous different commercial versions available. Basar [35] analyzed *B. neglecta* and *B. rivae* EO by isolating and identifying monoterpenes. The main constituents identified in *B. neglecta* were *α*-pinene (21.3%), *α*-thujene (21.3%), terpinen-4-ol (5.3%), Δ-3-carene (1.9%), sabinene (1.3%), p-cymene (11.8%), and verbenone (2.1%). The resin oil composition of *B. rivae* is strongly related to that of *B. neglecta,* which contains cara-2,4-diene (1.8%), *α*-thujene (2.9%), *α*-pinene (16.7%), p-cymene (3.2%), o-cymene (3.9%), Δ-3-carene (17.3%), and limonene (21.1%). Furthermore, multiple triterpenoid constituents were identified from the pyrolysate of *B. neglecta,* namely *β*-amyrin (0.7%), *α*-amyrin (9.1%), epi-*α*-amyrin (1.6%), *α*- and *β*-amyrin (3-,12-dien-α-amyrin) (3.4%), *β*-amyrenone (1.4%), and 3-,12-dien-*β*-amyrin (1.1%). Likewise, from the pyrolysate of *B. rivae*, *α*-amyrin (4.2%), 24-norursa-3,12-diene (18.7%), *β*-amyrin (0.9%), *β*-amyrenone (2.3%), *α*-amyrenone (2.8%), and epi-*β*-amyrin (0.9%) were discovered. In the resinous EO of *B. papyrifera,* Dekebo et al. [51] reported the following components: linalool (3.2%), *α*-pinene (2.6%), n-octanol (8.0%), limonene (6.5%), octyl acetate (56%), caryophyllene oxide (21%), n-hexyl acetate (1%), and *β*-elemene (29%). In a study conducted by Bekana et al. [52] on *B. neglecta* from the Wachile area, *α*-pinene (a monoterpene) and three triterpenoid components, *α*-amyrenone, *β*-amyrenone, and *α*-amyrin, were identified in the chromatogram of the resinous methanolic extract. At 6.33 min, a peak developed that was later determined to be *α*-pinene. The authors reported that *β*-amyrenone, *α*-amyrenone, and *α*-amyrin had retention times of 36.78, 38.20, and 38.93 min, respectively. In addition, *α*-pinene and two triterpenoid components, *α-* and *β*-amyrin, were detected in the chromatogram of a resinous methanolic extract of *B. rivae* from the Chereti area. Alpha-pinene, beta-amyrin, and alpha-amyrin were identified as components with retention times of 6.38, 37.47, and 38.13 min, respectively. One diterpene and three triterpenes were also identified in the methanol extract of *B. papyrifera*, whose resin was gathered in Ethiopia's northern regions (Metema, Metekel, and Humera areas). A chromatogram profile of the methanolic extract collected from the Humera region identified *β*-amyrenone, incensole acetate, *α*-amyrin, and *β*-amyrin as components with retention times of 24.83, 20.87, 26.25, and 24.95 min, respectively.

Analyses of three *Boswellia* species revealed high concentrations of monoterpenoids, diterpenes, and triterpenes in their methanol extract. *B. papyrifera* is distinct from the other species due to its octyl acetate, n-octanol, and incensole acetate content. However, there is insufficient evidence to conclude that *B. papyrifera* and the other two species (*B. rivae* and *B. neglecta*) differ so significantly in chemical composition that they fail to meet export standards [52].

Previous research has revealed that the high concentration of monoterpenes (such as *α*-pinene and D-limonene) in *BO* dramatically decreases the cellular viability of MCF-7, MDAMB-23, and T47D human breast cancer cell lines [53]. Similarly, it also diminishes the cellular viability of various cancer cell lines, including human bladder cancer J82 cells [54]. However, these cytotoxic effects do not significantly affect the cellular viability of noncancer cell lines such as human embryonic kidney (HEK)-293 cells, normal breast (MCF10-2A) cells, or normal bladder urothelial cells (UROtsa) [55]. *α*-Pinene stimulates apoptosis, the programmed cell death mechanism by which natural killer lymphocytes target and destroy cancer cells [56]. D-Limonene, by contrast, has numerous and direct pharmacological effects on cancer cells, including suppressing cancer cell proliferation and chemoresistance [57]. The EO of *Boswellia sacra* contains monoterpenoids, which in vitro research on *Pseudomonas aeruginosa*, *Propionibacterium acnes,* and *Staphylococcus aureus* reveals is antibacterial. FEOs also display potent antifungal activity against the yeasts *Malassezia furfur* and *Candida albicans* [58].

## 3. Diterpenes in *Boswellia sacra*

**Figure 2:**
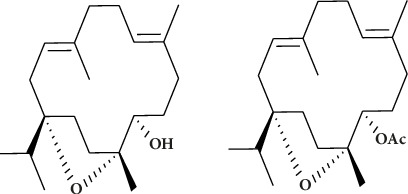
Structure of incensole (1) and incensole acetate (2) [60].

Several notable cembranes and cembranoids, including incensole (1) and incensole acetate (2), have been isolated. Ten years after Corsano and Nicoletti [59] reported isolating incensole, in 1977, Obermann [19] announced that incensole acetate had been isolated. Chemical alteration of incensole and its acetate, including the functionalization of olefinic bonds and the hydroxyl group at C-5, has added to the structural diversity already existing in nature, as displayed in Figure 2 [60].

The cembrane-type diterpenoids identified in frankincense, including incensole and its acetate, have anti-inflammatory, anxiolytic, antidepressant, and antitumor properties. Unfortunately, normal chemical changes in these scaffolds that could be used to generate novel and more effective anti-inflammatory medicines are lacking. Notably, most modern medications are themselves heterocyclic molecules. The greatest levels of incensole and its acetate have been found in *B. papyrifera*, raising serious concerns regarding the longevity of this species [61]. Furthermore, abnormally high concentrations of proinflammatory cytokines have been detected in the blood and cerebrospinal fluid (CSF) of people with schizophrenia [62]. Incensole and its acetate, which have anti-inflammatory properties, may therefore be useful in treating this condition [63].

**Figure 3:**
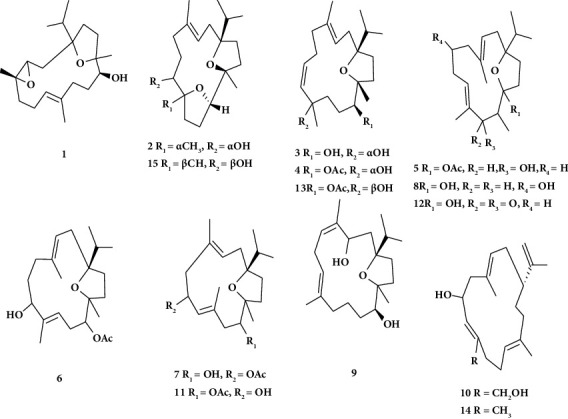
Structure of diterpenoids from *B. sacra* Flueck [64].

In an ongoing analysis of the 95% ethanolic extract from *Boswellia sacra,* Wang et al. [64] found fifteen cembranoids, including the previously unreported boscartins AL-AU (1–10), as shown in Figure 3. Several have neuroprotective or hepatoprotective properties. A phytochemical study of the gum resin of *Boswellia sacra* led to the extraction of 10 hitherto unreported cembrane-type diterpenes (boscartins AL-AU, 1–10) and five previously identified counterparts (11–15). Neuroprotective effects on cell viability in response to glutamate toxicity were similarly demonstrated by the *Boswellia sacra*–isolated diterpene boscartin AU (66) (68.4%) and the standard PHPB (68.9%) [64].

## 4. Sesquiterpenes in *Boswellia sacra*

**Figure 4:**
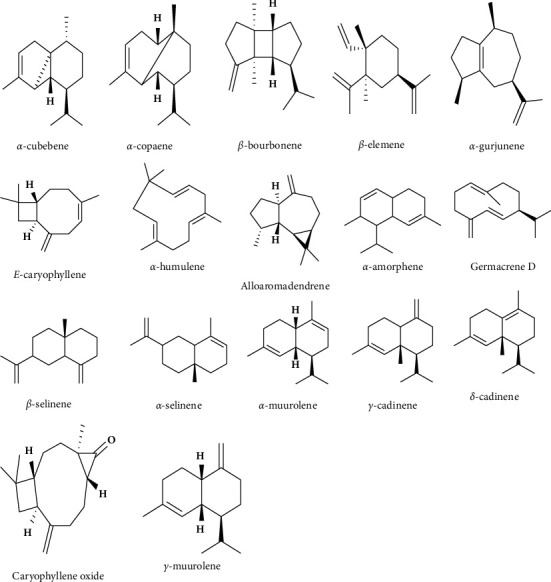
Structure of sesquiterpenes from the FEO of *B. sacra* resin [10].

The volatile oil contains various terpenes, including monoterpenes (as hydrocarbons, alcohols, and ketones) and sesquiterpenes, along with small amounts of diterpenes. Unsurprisingly, the composition of the oil varies depending on its environment, harvest, and geographical origin [31]. Numerous boswellic acids have been extracted from the oleo-gum resin of Arabian Indian and Somalian *Boswellia* species [22, 23, 32, 34]. The sesquiterpenes were identified as *α*-copaene (0.3%), *α*-cubebene (0.1%), *β*-bourbonene (0.1%), *α*-gurjunene (0.1%), *α*-humulene (0.2%), E-caryophyllene (0.9%), *α*-amorphene (0.1%), germacrene D (0.1%), *α*-selinene (0.1%), *α*-muurolene (0.1%), *β*-selinene (0.1%), *γ*-cadinene (0.1%), *γ*-muurolene (0.1%), and caryophyllene oxide (0.01%), as displayed in Figure 4 [10].

Niebler et al. [65] enriched and isolated Compounds 1 and 2 from the sesquiterpene fraction using semipreparative and analytical techniques. Distillation from bulb to bulb (or Kugelrohr) at a microscale is a useful technique for separating out trace levels of volatiles and semivolatiles. Following a thorough literature search for mass spectra of sesquiterpene ketones, the chemicals rotundone (1) [66] and mustakone (2) [67] were tentatively identified using their mass spectra and retention indices. Although *Boswellia sacra* frankincense is known to contain a wide range of sesquiterpenes [32, 68], this was the first evidence to suggest that oxygenated sesquiterpenes might be important contributors to the smell of frankincense.

## 5. Triterpenoids in *Boswellia sacra*

**Figure 5:**
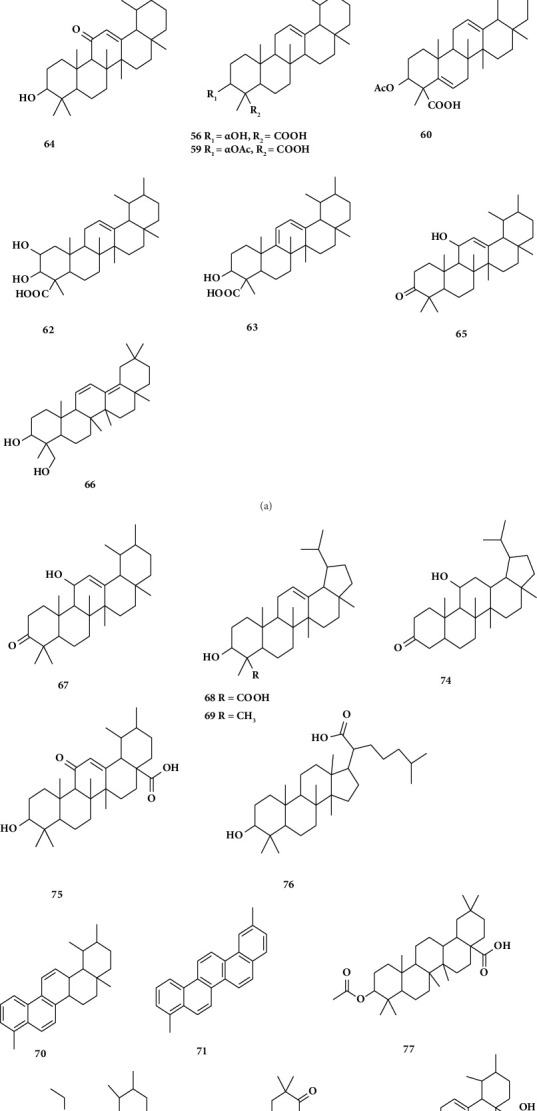
Structure of isolated triterpenoids from *B. sacra* [77].

Lupeolic acid, *α*- and *BA*s (54, 55, 56), and the corresponding O-acetyl derivatives (57, 58, and 59) are examples of triterpenoid chemicals found in *Boswellia sacra* that elucidate its chemical makeup [69]. Through an analysis of pentacyclic triterpenic acids, Ali et al. [70] identified two new O-acetyl derivatives: 3-acetoxylup-12:20-dien-24-oic acid (61) [71] and 3α-acetoxyurs-5:12-dien-24-oic acid (60) from Omani *Boswellia sacra* [70]. By pyrolyzing the resin of *Boswellia sacra and* then capturing the resulting smoke in a specially constructed device, Al-Harrasi et al. [72] identified one oleanane type (67) and one ursane type (66), along with lupeolic acid (68) and lupeol (69) (lupane-type triterpenoids). In the smoke-saturated water, two compounds, 1,2,4a,9-tetramethyl-1,2,3,4,4a,5,6,14b-octahydropicene (70) and 2,9 dimethyl picene (71), were obtained from the n-hexane extract. Tests of pyrolysate products (70) and (71) confirmed their ability to limit the proliferation of MDAMB-231 breast cancer cells [73]. From the Omani *Boswellia sacra* Flueck, Al-Harrasi et al. [74] reported the presence of three chemicals: the well-known compounds, papyriogenin B (73) and rigidenol (74), and the novel ursane-type triterpene, nizwanone (72). From a methanolic extract, triterpenoid compounds such as 3-O-acetyl-ursolic acid (78), 11-keto ursolic acid (75), and 3-O-acetyloleanolic acid (77) were found [75]. Likewise, seven more known chemicals, including triterpene [68], were identified from *Boswellia sacra* resin [76]. Structures of various triterpenoids from *Boswellia sacra* are depicted in Figure 5 [77].

## 6. Pharmaceutical Applications of Monoterpenoids

### 6.1. Antimicrobial Effects

Traditional medicine has long relied on *Boswellia sacra's* EO to treat bacterial and fungal infections. An in vitro examination of monoterpenoids in the EO revealed that *Pseudomonas aeruginosa*, *Staphylococcus aureus*, and *Propionibacterium acnes* were inhibited. Significant antifungal activity against *Malassezia furfur* and *Candida albicans* was also observed in FEOs. The antibacterial and antibiofilm efficacy in the oleoresin extract of *Boswellia sacra* against *Porphyromonas gingivalis* was also investigated [78]. Furthermore, the effectiveness of *Boswellia sacra* extract was tested against certain bacterial infections and germs that stimulate autoimmune diseases in humans when used in conjunction with standard antibiotics. The results revealed that when conventional antibiotics were combined with *Boswellia sacra* extracts, significantly greater activity was observed than in either component alone [79].

Against the fungi that cause strawberry rot, such as *Botrytis cinerea, Aspergillus niger*, and *Rhizopus stolonifer, Boswellia sacra* EO exhibits potential antifungal activity [80]. *Campylobacter jejuni* is a common cause of gastrointestinal illness and drug resistance. Antibiotic resistance in *C. jejuni* can be modulated by *α*-pinene [81]. A 512-fold decrease in the minimum inhibitory concentration (MIC) of ciprofloxacin, erythromycin, and triclosan has been recorded, demonstrating that *α*-pinene controls antibiotic resistance [82]. In a study comparing its activity against *Candida albicans* and both Gram-positive and Gram-negative bacteria, (+)-*β*-pinene was roughly 2–12 times more active than (+)-*α*-pinene [83]. The damaging effects of both on the membranes of infectious pathogens justify their usage as antibacterial agents [84]. Antitumor and antileukemia effects have also been demonstrated [85]. In addition, (+)-*α*-pinene exhibits antimalarial activity 250 times higher than that of (+)-*β*-pinene. In comparison with other chemicals such as vitamin C, terpenes display significantly higher levels of antioxidant activity [86].

### 6.2. Antitumor Activity

Defined by uncontrolled cell division, tumors are diseases of cell proliferation and differentiation. Mortality rates are highest for lung cancer, with 1.38 million people deaths per annum [87]. Terpenoid alpha-pinene is useful in the treatment of ovarian cancer, hepatocellular carcinoma, and N2a neuroblastoma due to its antitumor properties [88–90]. When used with the breast cancer medication paclitaxel, *α*- and *β*-pinene exhibit potent antitumor action [91]. An antiproliferative effect against breast cancer (MDAMB-231 and MCF cell) has been observed in an EO produced by hydrodistillation in which *α*- and *β*-pinene are particularly abundant [54].

### 6.3. Neuroprotective Activities

Neurodegenerative illnesses such as Alzheimer's and Parkinson's can be initiated by an oxidative imbalance. Brain health and the prevention of neurodegenerative diseases depend on free radical generation being kept to a minimum. Using mouse pheochromocytoma cells as a model, Porres-Martinez et al. [92] studied the effect of *α*-pinene on H_2_O_2_-induced oxidative stress [12] and found that *α*-pinene inhibits the generation of free radicals within cells [92].

### 6.4. Inhibitory Effect on the Growth of Endocarditis Disease

Bacterial and, less frequently, fungal infections can lead to endocarditis—an inflammation of the endocardium, the heart's inner lining. This is primarily caused by microorganisms from the genera *Streptococcus* and *Staphylococcus*, as well as *H. aphrophilus, Haemophilus parainfluenzae, H. paraphrophilus, Actinobacillus actinomycetemcomitans, Eikenella corrodens, H. influenzae, Kingella denitrifican*s, and *Cardiobacterium hominis*. One study examined the effects of *α*- and *β*-pinene on the progression of endocarditis. The recommended screening employed *Staphylococcus epidermidis, Staphylococcus aureus, Staphylococcus pyogenes,* and *Staphylococcus pneumoniae*. Both *α*- and *β*-pinene inhibited the growth of the aforementioned microorganisms*. S. aureus* was resistant to *α*- and *β*-pinene, and certain strains developed resistance to antibiotics (most notably gentamicin) [82].

### 6.5. Anti-Inflammatory and Analgesic Activities

The immune system's response to infection is inflammation. A study conducted to determine whether alpha-pinene inhibited inflammation in the peritoneal macrophages of male (C57BL/6) rats revealed that macrophage IL-6 and TNF-*α* production is inhibited by *α*-pinene, which also inhibits nitrite formation [93]. The primary components of FEO include *α*-pinene, (E)-*β*-ocimene, 1-octanol, *α*-thujene, linalool, limonene, and octyl acetate. These bioactive chemicals have powerful local anti-inflammatory and analgesic activities. The proposed method involved preventing inflammatory infiltrates caused by nociceptive stimuli and cyclooxygenase-2 (COX-2) overexpression [94]. *α*-Pinene therapy was observed to reduce nuclear factor (NF)-κB and mitogen-activated protein kinase (MAPK) pathway activation [93]. Furthermore, alpha-pinene compounds significantly reduced the proinflammatory expression of cytokines [95]. The suppression of IL-1*β*, TNF-*α*, nitric oxide, and MAPKs indicates that this active component has anti-inflammatory effects [96].

*α*-Pinene has proven useful in treating respiratory airway (both upper and lower) illnesses by reducing NF-κB translocation in THP-1 cells [85]. The anti-inflammatory effects of FEO are mediated through the downregulation of COX-2, suppression of NF-*κ*B transactivation, and reduction in TNF-*α* and IL-6 production [97]. The bioactive component thymol is responsible for FEO's anti-inflammatory properties [98, 99]. Several reports have revealed that carvacrol, a vigorous ingredient in FEO, can reduce inflammation by inhibiting the production of inflammatory cytokines such as IL-1*β* [100].

### 6.6. Acetylcholinesterase (AChE) Inhibition

Awadh Ali et al. [101] examined how EOs of *Boswellia* species can inhibit AChE. The EO of *B. socotrana* (59.3% inhibition) was more effective at blocking a 200 μg/mL dose of AChE than that of *B. elongata* (29.6% inhibition) and *B. ameero* (41.5% inhibition). This may be because *B. socotrana* oil contains (E)-2,3-epoxycarene and p-menth-1(7)-en-2-one, monoterpenoid skeletons known to stop AChE from working [102, 103]. Pulegone is a monoterpene from *Mentha spp.* with a p-menthane molecular structure that can prevent AChE with an IC50 of 890 μM [103].

### 6.7. COVID-19 and FEO

EOs have major chemical parts called monoterpenoids, sesquiterpenoids, and phenylpropanoids. These give the oils their medical effects, such as fighting inflammation, bacteria, free radicals, and cancer. As demonstrated in both lab tests and real-life clinical trials [104], EOs from some herbs excel at fighting the herpes simplex virus type 1, the Junin virus, the influenza virus, and the SARS coronavirus. One study reported that carvacrol and its isomer thymol, discovered in oregano, can stop HIV-1 cells from entering the body [105]. Another found that carvacrol interacts with the important protease protein (M pro), halting SARS-CoV-2 replication [106]. Thus, carvacrol might be an effective treatment for COVID-19 infection. Additionally, certain phenolic monoterpenoids, including carvacrol and thymol, may prevent a spike (S) glycoprotein from attaching to host cells [107]. Although evidence as to how anti-inflammatory treatments work in COVID-19 patients is lacking, FEO and its active ingredients, such as phenolic monoterpenoid (thymol and carvacrol), may serve as a potential treatment for future inflammatory problems related to COVID-19 [73]. However, it is as yet unclear whether carvacrol and its isomer thymol also stop the expression of angiotensin-converting enzyme 2 (ACE2) and M pro. Further research is required to determine what part of FEO is bioactive against COVID-19.

## 7. Conclusion and Future Research

This study revealed that *Boswellia sacra* is a significant medicinal plant. In addition to a high concentration of monoterpenoid ingredients, methanol extracts of *Boswellia* species contain diterpenes and triterpenes. The Dhofar region is rich in frankincense trees. The *Boswellia* extract and chemicals generated offer significant therapeutic potential for treating serious illnesses. Alpha-pinene, a key component of EO, has been lauded for its potent antitumor and antimicrobial properties. In a variety of tumor models, AKBA and other boswellic acids found in *Boswellia* gum resin exhibited significant antitumor activity. The overall potential of frankincense's active principle is substantial. *Boswellia sacra* has a wide variety of biologically active terpenoid chemicals, particularly monoterpenes, which are responsible for an array of biological functions. However, no information is currently available on the clinical efficacy of *Boswellia sacra*. This literature review therefore indicates that controlled clinical trials prior to medication development should be conducted on the memory-enhancing properties of *Boswellia sacra*.

## Figures and Tables

**Table 1:** Predominant compounds in the EO of reported *Boswellia* species.

*Boswellia* species	Predominant compounds	Percentage	Literature
*B. serrata*	Myrcene	38	35
*B. serrata*	*α*-Thujene	22.7–47.4	36
*B. serrata*	*α*-Thujene	29.3	37
*B. serrata*	*α*-Thujene	61.36	38
*B. carterii*	Duva-3,9,13-triene-1a-ol-5,8-oxide-1-acetate	21.4	31
*B. carterii*	Octanol acetate	45.2	39
*B. carterii*	*α*-Pinene	67.3	40
*B. sacra*	Limonene + *β*-phellandrene	6.2	40
*B. sacra*	*α*-Pinene	68.2	40
*B. rivae*	Limonene	1.1–19.6	41
*B. rivae*	*α*-Pinene	32.5–66.2	41
*B. rivae*	Octanol	17.8	37
*B. papyrifera*	Octyl acetate	57.1–65.7	41
*B. papyrifera*	Octyl acetate	63.5	37
*B. pirottae*	Trans-Verbenol	15.5	26
*B. pirottae*	Terpinen-4-ol	14.6	26

**Table 2:** Percentages of volatile oil in each class of compounds in *B. carterii* EO.

Class of compounds	%
Monoterpenes	85.0
Monoterpene hydrocarbons	79.3
Oxygenated sesquiterpenes	1.9
Oxygenated monoterpenes	5.7
Monoterpene ketones	1.0
Monoterpene esters	1.0
Monoterpene alcohols	3.7
Sesquiterpenes	12.3
Sesquiterpene alcohols	0.3
Sesquiterpene epoxides	1.6
Sesquiterpene hydrocarbons	10.4

**Table 3:** Essential compounds of *Boswellia sacra* found in different places around the Dhofar Mountain range (Salalah, Oman).

Compound	Mughsayl (*n* = 78)		Hasik (*n* = 10)	
	Mean (%)	Std. dev	Mean (%)	Std. dev
*α*-Pinene	81.4	2.68	60.4	5.91
Limonene	3.7	1.32	13.6	2.70
Camphene	2.1	0.24	1.6	0.19
Myrcene	1.1	1.16	11.4	3.11
Sabinene	1.7	0.91	2.5	0.76
p-Cymene	0.7	0.20	1.3	0.37

**Table 4:** Chemical composition of monoterpenes in volatile oil extracted from *Boswellia sacra*.

Peal no.	RT	Compound name	%
1	8.024	Tricyclene	0.11
2	8.229	*α*-Thujene	0.13
3	8.447	*α*-Pinene	61.05
4	8.908	Camphene	3.67
5	9.118	Verbenene	0.43
6	9.69	p-Cymene	0.25
7	9.798	Sabinene	0.09
8	9.877	*β*-Pinene	2.83
9	10.454	*β*-Myrcene	0.94
10	10.873	*α*-Phellandrene	0.16
11	11.073	*δ*-3-Carene	4.22
12	11.189	p-Cymenene	0.06
13	11.32	*α*-Terpinene	0.19
14	11.608	o-Cymene	3.57
15	11.743	D-Limonene	9
17	12.856	*γ*-Terpinene	0.35
24	17.586	Alloocimene	1.35
29	21.52	R(+)-limonene	0.11

## Data Availability

The data that support the findings of this study are available from the corresponding author upon reasonable request.
